# Low-Grade Versus Medium-Grade Nuclear Sclerotic Cataract Density Produces Identical Surgical and Visual Outcomes: A Prospective Single-Surgeon Study

**DOI:** 10.7759/cureus.11997

**Published:** 2020-12-09

**Authors:** Danny Lam, Helen Zhang, Neeranjali S. Jain, Ashish Agar, Ian C Francis

**Affiliations:** 1 Department of Ophthalmology, Sydney Hospital and Sydney Eye Hospital, Sydney, AUS; 2 Department of Ophthalmology, The University of New South Wales, Sydney, AUS; 3 Department of Ophthalmology, Prince of Wales Hospital, Sydney, AUS

**Keywords:** cataract surgery, nuclear sclerotic density, visual outcomes

## Abstract

Purpose

To determine whether the incidence of major complications and postoperative corrected distance visual acuity are comparable for surgery on low-grade versus medium-grade nuclear sclerotic cataracts.

Design

This was a prospective, consecutive, single-surgeon, no-exclusion study of 1025 cataract cases with one-month follow-up.

Methods

Patients were divided into two cohorts according to the nuclear sclerosis grade at presentation, as classified using the Lens Opacities Classification System (LOCS) III. Cohort A, representing low-grade nuclear sclerotic cataracts (grades 1-2), consisted of 739 eyes, while Cohort B, representing medium-grade nuclear sclerotic cataracts (grades 3-6), consisted of 286 eyes.

Results

There was no significant difference in major intraoperative or postoperative complications (p>0.999) between Cohorts A and B. The mean logMar preoperative corrected distance visual acuity (CDVA) in Cohort A was 0.245 as compared with 0.346 in Cohort B (p<0.001). There was no significant difference between cohorts for postoperative CDVA at one day (-0.168 versus -0.118; p=0.070), one week (-0.180 versus -0.147; p=0.405), or one month (-0.185 versus -0.161; p=0.569).

Conclusions

There was no significant difference in the incidence of operative complications or postoperative CDVA between the cohorts. These findings suggest that, in experienced hands, surgery for medium-grade nuclear sclerotic cataracts is equally effective and safe as compared with that for low-grade nuclear sclerotic cataracts.

## Introduction

A cataract is the most common cause of visual impairment [1] and blindness [2] worldwide. In Australia, the estimated prevalence of cataracts has been projected to rise by 63% from 2001 to 2021 [1]. As the number of patients affected by cataract rises, the optimal management of this condition becomes increasingly important [3]. Indeed, knowing the best time to operate may be of importance in attempting to achieve not only the best surgical outcomes (SOs) and visual outcomes (VOs) but also giving patients the most effective and enduring vision for their visual needs.

Improvements in the technology available to cataract surgeons have led to better surgical outcomes, and many barriers to performing surgery earlier in the disease process have been overcome [4-5].

Some surgeons consider that performing cataract surgery earlier rather than later is better for the patient, especially in the setting of the more rapid progression of higher grades of nuclear sclerosis (NS) and posterior subcapsular cataracts [6]. However, the literature does not provide a definitive compendium of reasoning for earlier cataract surgery. Certainly, there has been very little research on visual outcomes, as measured by corrected distance visual acuity (CDVA), in earlier versus later surgery [7].

The Royal College of Ophthalmologists states the aims of modern cataract surgery to be: (1) restoring vision, (2) achieving the desired refractive outcome, (3) improving quality of life, and (4) ensuring patient safety and satisfaction [8].

The premise for the current study was to assess whether earlier cataract surgery necessarily equated to better SOs and VOs. It was hypothesized that patients with higher grades of NS would exhibit poorer preoperative and postoperative CDVA and hence VOs. Furthermore, it was hypothesized that patients with higher grades of NS would sustain higher rates of major intraoperative and postoperative complications.

## Materials and methods

This was a prospective, consecutive, no-exclusions study of a single experienced cataract surgeon’s patients, recruited over four years. All surgery was undertaken at Chatswood Private Hospital, a dedicated private ophthalmic day surgery center in suburban Sydney, Australia. This study was conducted in accordance with the National Statement on Ethical Conduct in Human Research, and consistent with the principles that have their origin in the Declaration of Helsinki. Ethics approval was granted by the University of New South Wales, Australia.

Four aims were developed for this study:

1. To compare preoperative CDVA of patients with low versus medium-grade NS cataracts

2. To compare postoperative CDVA of patients with low versus medium grade NS cataracts

3. To compare the incidence of SOs between patients with low versus medium grade NS cataracts undergoing cataract surgery

4. To determine whether earlier cataract surgery resulted in more favorable postoperative VOs in patients with low versus medium-grade NS cataracts

All patients in the cohort were selected for surgery on the basis that their visual function could be improved by cataract surgery. Their fitness for surgery was determined by an adequate American Society of Anesthesiologists (ASA) grading [9]. No patient was excluded based on any aspect of his or her cataract, including pupillary dilatation, pseudoexfoliation, the severity of refractive errors, and posterior subcapsular cataracts (PSCC). Each case was treated on its own merits, with the overall aim of achieving an uncomplicated surgical procedure and a postoperative CDVA log of the minimal angle of resolution (logMAR) of at least -0.1.

In this study, the Lens Opacities Classification System (LOCS) III cataract grading schema [10] was used to grade the degree of NS in order to determine how early or late in the disease process the surgery was performed. This is contingent upon the fact that as NS becomes more advanced over time, the NS grade usually increases [11]. Grades 1 and 2 cataracts were considered low grade (Cohort A) and grades 3-6 were considered medium grade (Cohort B). While high degrees of PSCC were encountered, in the current study, only the degree of NS was considered as the defining factor of cataract severity.

Preoperative and postoperative assessment

A complete ophthalmological examination, including CDVA assessment, was conducted preoperatively in each patient. Postoperative reviews, including CDVA, were carried out at one day, one week, and one month. Where applicable, CDVA assessment was assisted by automated refraction. Retinoscopy and subjective refraction were employed when automated refraction failed due to dense NS preoperatively, high grades of PSCC, or small pupils. All intraoperative and postoperative complications were documented. The NS grade was determined by comparing each crystalline lens assessed with a set of standard images on the LOCS III scale, aiming to grade nuclear color and nuclear density from 1 to 6 [10].

Surgical method

Standard phacoemulsification cataract surgery was performed using the Alcon Infiniti® system (Alcon, Geneva, Switzerland), employing the phaco stop and chop technique [12]. Ophthalmic anesthesia was carried out by a specialist anesthetist utilizing assisted topical or assisted local anesthesia [13-14]. Mainport and sideport clear corneal incisions were made. Unless otherwise indicated, the Alcon SN60WF® or SN6AT® hydrophobic acrylic toric or non-toric intraocular lens was implanted with wound assistance.

Statistical analysis

All data were collected prospectively and analyzed using the Statistical Package for the Social Sciences (SPSS) for Windows® (version 19.0, IBM). p-values less than 0.05 were considered statistically significant. Multiple linear regression analysis was used to test for differences in CDVA between the two cohorts, adjusting for age, gender, and preoperative CDVA. Postoperative changes in CDVA for each patient were compared with his or her preoperative CDVA. Fisher’s exact test was applied to analyze the relationship between NS grade and the incidence of surgical complications.

CDVA was measured using the Snellen visual acuity scale. Conversions were made to the logMAR scale in order to run the above tests, for which a linear scale for computation of arithmetic means was required.

## Results

Over the study period, 1025 prospective, consecutive surgical cases were documented. Females comprised 60.9% of the total cohort. The patient ages ranged from 23 to 94 years, with a mean age of 71 years. NS grades were documented in all cases, with 70.1% of the cohort having grade 2 NS. Table 1 illustrates demographics and the spread of patients across NS grades. Cohort A consisted of 739 patients and Cohort B of 286 patients.

**Table 1:** Patient Demographics and Nuclear Sclerotic Grades NS, nuclear sclerosis; SD, standard deviation

Total number of patients		1025
Female gender (% of patients)		624 (60.9%)
Average age (years +/- SD)		70.9 +/- 8.9
Number (% of patients) with NS:	Grade 1	20 (2.0%)
	Grade 2	719 (70.1%)
	Grade 3	269 (26.2%)
	Grade 4	14 (1.4%)
	Grade 5	3 (0.3%)
	Grade 6	0 (0%)

Preoperative and postoperative CDVAs (at one day and one week) were documented in 100% of cases. One-month postoperative CDVA values were documented in 99.5% of cases (all but five patients from Cohort A). Adjusting for age and gender, patients in Cohort A had better CDVA preoperatively than those in Cohort B (Table 2). Expressed in logMAR, the mean preoperative CDVA was 0.245 in Cohort A and 0.346 in Cohort B (p<0.001).

**Table 2:** Preoperative CDVA CDVA, corrected distance visual acuity; SD, standard deviation

	Cohort A	Cohort B	P-value
Number	739	286	
Preoperative CDVA (logMAR +/- SD)	0.245 +/- 0.159	0.346 +/- 0.226	<0.001

Adjusting for age, gender, and preoperative CDVA, there was no significant difference between the two cohorts in postoperative CDVA at one day (-0.168 in Cohort A versus -0.118 in Cohort B; p=0.070), one week (-0.180 versus -0.147; p=0.405), or one month (-0.185 versus -0.161; p=0.569). It was considered that the slightly better mean postoperative CDVAs observed in Cohort A were unlikely to have clinical significance (Table 3).

**Table 3:** Postoperative CDVA and Major Operative Complications CDVA, corrected distance visual acuity; SD, standard deviation

	Cohort A	Cohort B	P-value
Number	739 (734 at 1 month)	286	
Postoperative CDVA			
Postoperative CDVA (logMAR +/- SD) at 1 Day	- 0.168 +/- 0.108	- 0.118 +/- 0.202	0.070
Postoperative CDVA (logMAR +/- SD) at 1 Week	- 0.180 +/- 0.090	-0.147 +/- 0.184	0.405
Postoperative CDVA (logMAR +/- SD) at 1 Month	- 0.185 +/- 0.066	- 0.161 +/- 0.158	0.569
Major intraoperative and postoperative complications			
Number with posterior capsule tears (incidence)	3 (0.41%)	1 (0.35%)	>0.999
Number with cystoid macular edema (incidence)	2 (0.27%)	0 (0.00%)	>0.999
Total (incidence)	5 (0.50%)	1 (0.35%)	>0.999

There was no significant difference in the incidence of major intraoperative and postoperative complications between patients with low versus medium NS grades (p>0.999). The intraoperative and postoperative complications encountered were posterior capsule tear (PCT) and cystoid macular edema (CMO). There were three occurrences of PCT and two of CMO in Cohort A while Cohort B had one instance of PCT and no CMO (Table 3). There were no cases of postoperative endophthalmitis.

## Discussion

This study assessed and compared the intraoperative and postoperative complications and VOs of patients who underwent cataract surgery for different grades of NS. As expected, this study demonstrated a statistically significant difference in preoperative CDVA between Cohorts A and B, reflective of differing grades of NS cataracts. There were few high-grade NS cataracts, with only three patients having grade five cataracts, and no patients having grade six cataracts. This is unsurprising, given that the study was carried out in a suburban ophthalmology center in a developed country, where very high-grade NS cataracts are less likely to present. Thus, this study presents a comparison of postoperative SOs and VOs between patients with low and medium-grade NS cataracts.

There were no significant differences between the cohorts in postoperative CDVA at one day (p=0.070), one week (p=0.405), or one month (p=0.569). Furthermore, there was no significant difference in major intraoperative and postoperative complications between the two cohorts. It should be noted that this low incidence of major operative complications may have been associated with the surgeon’s experience and the use of standardized modern surgical techniques [15-16].

Importantly, these findings should not take precedence over patient factors that may mandate earlier surgery, including health and psychosocial status. It is known that cataract surgery has a major quality-of-life benefit, especially in the elderly [17]. Studies indicate that poor visual acuity is one of the most important modifiable predictors of falls [18], particularly following hip fracture surgery [19]. Furthermore, patients with posterior subcapsular and some NS cataracts are subject to glare. This is a notable concern for patients in bright sunlight or when faced with car headlights while driving at night. PSCCs may also progress relatively quickly [6]. Therefore, the difference in preoperative CDVA in our study (mean CDVA of 0.245 in Cohort A versus 0.346 in Cohort B; p<0.001) is likely to have clinical significance, affecting patients’ activities of daily living (ADL).

First eye cataract surgery may reduce falls risk in visually impaired patients by 34% [20]. In addition, marked improvements in ADL participation and patient satisfaction regarding vision have been exhibited following bilateral cataract surgery [21]. In terms of earlier versus later surgery, waiting more than six months for surgery may result in lower quality of life and an increased risk of falls. In contrast, expedited surgery has been associated with reduced rates of falls, fractures, anxiety, and depression [22]. Other potential benefits of undergoing surgery earlier include better health and younger age, allowing patients to undergo surgery when health comorbidities are minimized, with an increased number of years spent with optimal vision [23].

The authors, therefore, recommend cataract surgery if a patient becomes visually symptomatic in terms of deteriorating visual acuity for reading or distance, reduced vision in glare, or in patients with dementia where imminent institutionalization may be postponed by improved visual function. Shallowing of the anterior chamber due to an enlarging cataract, possibly with increasing intraocular pressure, or progressive pseudoexfoliation syndrome, while not documented in our study, may also be an indication for earlier surgery. However, the results in this study indicate that, in the hands of an experienced surgeon, SOs and VOs are not influenced by the NS grade at the time of surgery. Other studies have also shown that improvements in postoperative VOs are greatest in patients with worse preoperative CDVA [24]. However, patients with less improvement after surgery were more likely to have a better preoperative visual function since there is relatively less room for improvement [24]. Thus, when pertinent indications for surgery are not present, earlier surgery may not be justified. This information may prove useful when planning surgery and when reassuring patients who may face long waiting lists.

A limitation of this study is the relatively small number of cataracts of NS Grade 4 and above in the population studied. This may reflect clinician awareness that delaying surgery could allow the deterioration of visual acuity and, as a result, quality of life. It may also reflect the ease of access to cataract surgery in the study setting. Further studies can assess and compare the outcomes after surgery of higher grade cataracts (Grades 4-6), especially as cataracts of higher grade may require urgent cataract surgery or the use of a different surgical technique. Another limitation is the potential statistical bias associated with the adjustment for preoperative CDVA, as it is likely to be correlated with the NS grade. Additionally, patients were not specifically followed up as part of the study beyond one month for CDVA assessment, nor was their quality of life evaluated, as these outcomes have already been documented in the literature.

## Conclusions

This study demonstrated that CDVA was significantly better preoperatively in the patient group with lower grade NS than the group with more advanced cataracts. Cataract surgery was shown to be equally effective in restoring vision in patients with low versus medium-grade nuclear sclerotic cataracts. Furthermore, there was no significant difference between cohorts in the incidence of major intraoperative and postoperative complications.

Ideally, cataract surgery should be timed early enough in the disease process to maximize surgical safety, restore vision, and improve long-lasting quality of life and patient satisfaction. Indeed, this study demonstrates that when performed by an experienced surgeon, phacoemulsification for more advanced cataracts is equally effective in improving patient VOs compared with surgery for earlier cataract grades. This indicates that potentially superior VOs or lower complication risk alone may not justify earlier surgery. However, other patient factors, including diminished visual performance, progressive shallowing of the anterior chamber, and medical and psychosocial comorbidities, should be considered when determining the optimal timing for cataract surgery.

## References

[1] Rochtchina E, Mukesh BN, Wang JJ, McCarty CA, Taylor HR, Mitchell P (2003). Projected prevalence of age-related cataract and cataract surgery in Australia for the years 2001 and 2021: pooled data from two population-based surveys. Clin Exp Ophthalmol.

[2] Resnikoff S, Pascolini D, Etya'ale D, Kocur I, Pararajasegaram R, Pokharel GP, Mariotti SP (2004). Global data on visual impairment in the year 2002. Bull World Health Organ.

[3] Taylor HR, Vu HTV, Keeffe JE (2006). Visual acuity thresholds for cataract surgery and the changing Australian population. Arch Ophthalmol.

[4] Keeffe JE, Taylor HR (1996). Cataract surgery in Australia 1985-94. Aust N Z J Ophthalmol.

[5] Hahn U, Krummenauer F, Kölbl B (2011). Determination of valid benchmarks for outcome indicators in cataract surgery: a multicenter, prospective cohort trial. Ophthalmology.

[6] Obara Y (2005). Timing of cataract surgery. Japan Med Assoc J.

[7] Wang SB, Liu Y, George A, Francis IC (2016). Rethinking cataract surgery benchmarking, key performance indicators and maintaining professional standards in Australia. Clin Exp Ophthalmol.

[8] (2010). Cataract surgery guidelines. page 3.

[9] Jones HJS, Cossart LD (1999). Risk scoring in surgical patients. Br J Surg.

[10] Chylack LT Jr, Wolfe JK, Singer DM (1993). The Lens Opacities Classification System III. Arch Ophthalmol.

[11] Magalhaes FP, Costa EF, Cariello AJ (2011). Comparative analysis of the nuclear lens opalescence by the Lens Opacities Classification System III with nuclear density values provided by Oculus Pentacam: a cross-section study using Pentacam Nucleus Staging software. Arq Bras Oftalmol.

[12] Koch PS, Katzen LE (1994). Stop and chop phacoemulsification. J Cataract Refract Surg.

[13] Figueira EC, Sharma NS, Ooi JL (2009). The Lanindar test: a method of evaluating patient suitability for cataract surgery using assisted topical anaesthesia. Eye (Lond).

[14] Francis IC, Schumacher RS, Haylen MJ (1987). Assisted local anaesthesia for cataract surgery (ALACS). Aust N Z J Ophthalmol.

[15] Park DY, Walkden A, De Klerk TA (2016). Effect of cataract surgery training on operating room productivity: how long trainees take. J Cataract Refract Surg.

[16] Syed ZA, Moayedi J, Mohamedi M (2015). Cataract surgery outcomes at a UK independent sector treatment centre. Br J Ophthalmol.

[17] Ishii K, Kabata T, Oshika T (2008). The impact of cataract surgery on cognitive impairment and depressive mental status in elderly patients. Am J Ophthalmol.

[18] Ivers RQ, Cumming RG, Mitchell P, Attebo K (1998). Visual impairment and falls in older adults: the Blue Mountains Eye Study. J Am Geriatr Soc.

[19] Yau DY, Chung RK, Pang MYC (2013). Knee muscle strength and visual acuity are the most important modifiable predictors of falls in patients after hip fracture surgery: a prospective study. Calcif Tissue Int.

[20] Karlsson MK, Magnusson H, von Schewelov T, Cöster M, Rosengen BE (2013). Prevention of falls in the elderly: a review. Osteoporos Int.

[21] Harrer A, Gerstmeyer K, Hirnschall N, Pesudovs K, Lundström M, Findl O (2013). Impact of bilateral cataract surgery on vision-related activity limitations. J Cataract Refract Surg.

[22] Hodge W, Horsley T, Albiani D (2007). The consequences of waiting for cataract surgery: a systematic review. CMAJ.

[23] Lundström M, Stenevi U, Thorburn W (2002). The Swedish National Cataract Register: a 9‐year review. Acta Ophthalmol Scand.

[24] Saw SM, Tseng P, Chan WK, Chan TK, Ong SG, Tan D (2002). Visual function and outcomes after cataract surgery in a Singapore population. J Cataract Refract Surg.

